# Genomic analysis of five antibiotic-resistant bacteria isolated from the environment

**DOI:** 10.1128/mra.00751-24

**Published:** 2024-08-27

**Authors:** T. J. Bedore, Girish Kumar, Cherish McIntyre, Anishka Alvarez, Ariel Leslie, Au'Nisha Snead, André O. Hudson

**Affiliations:** 1 Thomas H. Gosnell School of Life Sciences, Rochester Institute of Technology, Rochester, New York, USA; 2 Rochester Prep High School, Rochester, New York, USA; Queens College Department of Biology, Queens, New York, USA

**Keywords:** *Bacillus*, *Atlantibacter*, *Pantoea*, *Enterococcus*, *Pseudomonas*, antibiotic resistance

## Abstract

Our study presents the whole-genome sequences and annotation of five bacteria isolates, each demonstrating distinct antibiotic resistance. These isolates include *Bacillus paranthracis* RIT 841, *Atlantibacter hermanii* RIT 842, *Pantoea leporis* RIT 844, *Enterococcus casseliflavus* RIT 845, and *Pseudomonas alkylphenolica* RIT 846, underscoring the importance of understanding antimicrobial resistance.

## ANNOUNCEMENT

Antimicrobial resistance (AMR) is not just a local or regional concern but a pressing global health problem. It is responsible for approximately 5 million deaths annually and is projected to escalate to 10 million by 2050 (1, 2). The misuse of antibiotics has been a significant driver of this crisis (3). This alarming rise in AMR necessitates a more judicious use of antibiotics, emphasizing positive culture results to guide antibiotic choices (4, 5). The genomic information gathered from AMR isolates is crucial for unraveling the mechanisms that underlie AMR.

Environmental samples were collected from various locations at Rochester Institute of Technology campus (Table 1) and placed in sterile 50-mL conical tubes. The samples were incubated in Tryptic Soy Broth (TSB) for 7 days at 25°C with shaking at 200 rpm. Serial dilutions (10^−1^ to 10^−10^) were prepared in TSB, and 100 µL of each dilution was plated on tryptic soy agar plates and incubated for 72 h at 25°C.

**TABLE 1:** Genome annotation information for the five antibiotic-resistant bacterial samples, with assembly metrics based on contig sizes greater than 500 bp

Sample	Sample source	Total # of reads	SRA accession	Assembly accession	Assigned taxonomy	Assembly size (bp)	Coverage (X)	# of contigs	N50	Assembly GC content (%)	# of genes	# of rRNA	# of tRNA
RIT_841	Soil	5,196,202	SRS21837156	JBEWWD000000000	*Bacillus paranthracis*	5,450,518	230	90	205,365	35.28	5,770	5	73
RIT_842	Rock	4,141,470	SRS21837157	JBEWWE000000000	*Atlantibacter hermannii*	4,347,768	225	37	236,652	54.19	4,123	4	68
RIT_844	Pinecone	2,008,300	SRS21837158	JBEWWF000000000	*Pantoea leporis*	4,839,527	97	22	737,083	54.89	4,467	6	69
RIT_845	Rock	2,226,178	SRS21837159	JBEWWG000000000	*Enterococcus casseliflavus*	3,406,239	177	14	379,111	42.93	3,245	3	52
RIT_846	Soil	2,921,434	SRS21837160	JBEWWH000000000	*Pseudomonas alkylphenolica*	5,651,020	143	54	245,649	59.68	5,270	2	64

**Fig 1:**
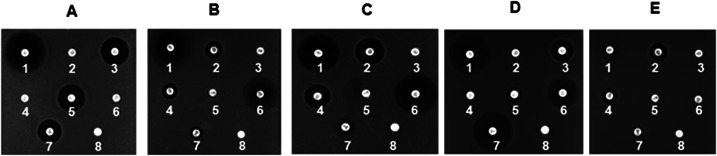
Antibiotic resistance screening of identified isolates *Bacillus paranthracis* RIT 841 (**A**), *Atlantibacter hermannii* RIT 842 (**B**), *Pantoea leporis* RIT 844 (**C**), *Enterococcus casseliflavus* RIT 845 (**D**), and *Pseudomonas alkylphenolica* RIT 846 (**E**). (1) Chloramphenicol, 30 mg/mL; (2) polymyxin B, 300 IU; (3) vancomycin, 30 mg/mL; (4) colistin sulfate, 10 mg/mL; (5) clindamycin, 2 mg/mL; (6) SXT, 25 mg/mL; (7) rifampicin, 5 mg/mL; and (7) negative control (10 µL of methanol).

Selected colonies (three per sample) based on color, morphology, shape, and texture were cultured in liquid TSB for 48 h. The isolates were tested against seven antibiotics (chloramphenicol, 30 mg/mL; polymyxin B, 300 IU; vancomycin, 30 mg/mL; colistin sulfate, 10 mg/mL; clindamycin, 2 mg/mL; sulfamethoxazole/trimethoprim (SXT), 25 mg/mL; and rifampicin, 5 mg/mL) using a disk susceptibility/resistance assay, which included a negative control (methanol, 10 µL) applied to a paper disk placed on TSB agar inoculated with bacterial cultures and incubated 48 h at 25°C (6). Isolates resistant to two or more antibiotics were chosen for genomic analyses. Isolate RIT 841 exhibited increased resistance to polymyxin B, colistin sulfate, and trimethoprim/sulfamethoxazole (Fig. 1A). Isolate RIT 842 exhibited increased resistance to vancomycin and clindamycin, with slight susceptibility to polymyxin B, colistin sulfate, and rifampicin (Fig. 1B). Isolate RIT 844 showed increased resistance to vancomycin and clindamycin, and slight susceptibility to rifampicin (Fig. 1C). Isolate RIT 845 exhibited increased resistance to polymyxin B, colistin sulfate, and slight susceptibility to clindamycin (Fig. 1D). Isolate RIT 846 exhibited increased resistance to five antibiotics, chloramphenicol, vancomycin, clindamycin, trimethoprim/sulfamethoxazole, and rifampicin, with a slight susceptibility to the remaining two antibiotics, polymyxin B and colistin sulfate (Fig. 1E).

Following the manufacturer’s protocol, DNA was extracted from isolates 25 mL of broth culture using the bacterial genomic DNA isolation kit (Sigma-Aldrich, USA). Libraries for sequencing were prepared with the Nextera XT library preparation kit (Illumina Inc., USA) per the manufacturer’s guidelines. Sequencing was conducted on an Illumina MiSeq platform (Illumina, San Diego, CA, USA) using the V3 Kit (2 × 300 cycles).

Default parameters were used for all the software unless otherwise specified. After quality control processing with fastp v0.23.2 (8), reads were assembled using SPAdes v3.15.4 (7). Quality assessment was evaluated by QUAST (9), and genome annotation was performed using National Center for Biotechnology Information Prokaryotic Genome Annotation Pipeline v. 6.7 (10). These isolates were identified as *Bacillus paranthracis* RIT 841, *Atlantibacter hermanii* RIT 842, *Pantoea leporis* RIT 844, *Enterococcus casseliflavus* RIT 845, and *Pseudomonas alkylphenolica* RIT 846.

## Data Availability

Table 1 presents the whole-genome assemblies, Sequence Read Archive (SRA), and accession numbers of the bacterial genomes, which can be downloaded from GenBank and SRA, respectively.
